# Comparison of the incidence, nature and cause of injuries sustained on dirt field and artificial turf field by amateur football players

**DOI:** 10.1186/1758-2555-3-3

**Published:** 2011-02-09

**Authors:** Ramin Kordi, Farajollah Hemmati, Hamid Heidarian, Vahid Ziaee

**Affiliations:** 1 The Sports Medicine Research Centre, Tehran University of Medical Sciences, Tehran, Iran; 2 Department of Sports and Exercise Medicine, Faculty of Medicine, Tehran University of Medical Sciences, Tehran, Iran

## Abstract

**Background:**

Data on the incidence, nature, severity and cause of match football injuries sustained on dirt field are scarce. The objectives of this study was to compare the incidence, nature, severity and cause of match injuries sustained on dirt field and artificial turf field by amateur male football players.

**Methods:**

A prospective two-cohort design was employed. Participants were 252 male football players (mean age 27 years, range 18-43) in 14 teams who participated in a local championship carried on a dirt field and 216 male football players (mean age 28 years, range 17-40) in 12 teams who participated in a local championship carried on a artificial turf field in the same zone of the city. Injury definitions and recording procedures were compliant with the international consensus statement for epidemiological studies of injuries in football.

**Results:**

The overall incidence of match injuries for men was 36.9 injuries/1000 player hours on dirt field and 19.5 on artificial turf (incidence rate ratio 1.88; 95% CI 1.19-3.05).

Most common injured part on dirt field was ankle (26.7%) and on artificial turf was knee (24.3%). The most common injury type in the dirt field was skin injuries (abrasion and laceration) and in the artificial turf was sprain and ligament injury followed by haematoma/contusion/bruise.

Most injuries were acute (artificial turf 89%, dirt field 91%) and resulted from player-to-player contact (artificial turf 59.2%, dirt field 51.4%).

Most injuries were slight and minimal in dirt field cohort but in artificial turf cohort the most injuries were mild.

**Conclusions:**

There were differences in the incidence and type of football match injuries sustained on dirt field and artificial turf.

## Background

Football (soccer) matches traditionally take place on natural grass, although different playing surfaces are used including sand, dirt, clay, concrete, asphalt, and hardwood. In dray counties maintaining a natural grass surface is expensive; therefore in this area especially in parts of Asian and Africa most football fields are dirt fields (DFs) (bare earth) and many football players, especially amateur ones, play on DFs in these regions. Another substitute in this area could be artificial turf field (ATF). In recent years the playing surfaces of amateur football players have begun being replaced by artificial grass in some counties[1].

The forces transmitted to football player tissues are varied on different surfaces. Therefore, injury frequency and injury pattern in football might be varied among players who play on different surfaces. It has been suggested that changes in surfaces might have effects on the performance and injury pattern of the sport[2,3]. Two main factors which might affect surface related football injuries reported to be the stiffness of a surface and the friction between surface and shoe[2,3]. DFs are generally not only stiff but also high friction surfaces. Moreover these fields might be uneven. These might lead to a higher and different type of injuries on diet field compare to other football fields.

Some studies have been evaluated the risk of football injuries on artificial turf (AT) and natural grass. These studies have shown conflicting results, although in general the range of outcomes has been of equivalent risk for injuries on AT and natural grass[4-7].

A few studies evaluated incidence of football injuries on other types of surfaces that show conflicting results. Engebretsen and Kase (1987) [2,8] investigated 16 Norwegian football teams over a two year period. They reported an injury rate of 20 per 1000 hours on gravel surface that was lower than the reported injury rate on AT.

McGrath and Ozanne-Smith also cited a study [2] in which incidence of injury on 230 football fields during 380,000 playing hours was investigated (1985). The injury incidence was reported as 2.6/1000 hours on grass, 1.8 on gravel and 0.4 on AT.

Arnason, et al (1996) [9] reported that significantly more injuries occurred on AT than on grass or gravel in correlation to number of hours in games and practices among Icelandic elite football players. In contrast, Ekstrand and Nigg (1989) [3] in their review on football surfaces, cited a 2-year study on the first artificial football surface in Sweden. They reported no difference in the incidence of injury between artificial, gravel or grass surfaces.

To our knowledge no study has evaluated the incidence, nature, severity and cause of match injuries sustained on dirt field (DF). The objectives of this study were to evaluate and to compare the incidence, nature, severity and cause of match injuries sustained on DF and ATF by amateur male football players.

## Methods

A prospective two-cohort design was employed in this study. Participants were 252 male non professional football players (mean age 27 years, range 18-43) in 14 teams who participated in a local (zone) championship carried on a DF during 13 weeks (91 matches) (first cohort), and 216 male non professional football players (mean age 28 years, range 17-40) in 12 teams who participated in a local championship carried on a ATF (Second Generation) during 11 weeks (66 matches) (second cohort). There are numerous football DFs in Tehran. Council of Tehran have recently started to cover some of these fields by AT. This made an opportunity to compare the injuries incidence rate of players who play on dirt and AT fields in two near zones which are in a same area in Tehran and are in similar athletic performance levels. Football dirt fields in Iran are football fields on bare earth that just have been leveled and not covered by any material such as clay or sand. Normally no maintenance activity is needed for these fields except for making re-flat the field every several years.

The incidence, nature, severity and cause of match injuries were recorded prospectively during the leagues. Both leagues were conducted during four months from mid July to mid November 2008. Injury definitions and recording procedures were compliant with the international consensus statement for epidemiological studies of injuries in football[10]. Two physicians recorded details of the diagnosis, severity and cause of all match injuries. One physician was present in each match that was carried out on either ATF or DFs. Match exposures were recorded by the physicians. The number of days lost from training and match play was used to define the severity of an injury. Match exposures (player hours) of all subjects were recorded.

An injury is defined as " any physical complaint sustained by a player that results from a football match, irrespective of the need for medical attention or time loss from football activities"[10]. We do not included problems other than physical complaints including illnesses and diseases.

The study procedure was approved by the Tehran University of Medical Sciences Ethics Committee.

### Data analysis

Incidences of match injuries on both artificial turf and dirt fields were reported as the number of injuries/1000 player match hours with 95% confidence interval (CI). Rate ratio was calculated to compare the ratio of injuries incidences that were occurred in two cohorts.

The differences between the injuries incidences were considered significant when the p value of the two-sided z test for the comparison of rates was less than 0.05.

Version 10 of "Data Analysis and Statistical Software" was used for data analysis.

## Results

Demographic characteristics of the subjects including player positions and leg dominancy are presented in Table 1.

**Table 1:** Demographic characteristic of the players who played on dirt field (DF) and artificial turf field (ATF).

	DF (95% CI)	ATF (95% CI)
Number of cases	252	216

Age(years)	27.04(26.50-27.58)	27.96(27.36-28.56)

Mean weight(kg)	72.79(72.01-73.58)	73.28(72.55-74.00)

Mean height(cm)	171.85(171.27-172.43)	172.31(171.72-172.90)

**% of dominant leg**		

• left	13(9-18)	13(8-17)

• right	70(62-74)	68(61-74)

• bilateral	17(12-22)	19(14-24)

**% of player position**		

• goalkeeper	14(9-18)	15(10-19)

• defender	34(27-39)	27(20-32)

• midfielder	33(27-38)	35 (28-41)

• forward	19(14-24)	23(17-28)

The overall incidence of match injuries for subjects was 36.9 injuries/1000 player hours on DF and 19.5 on ATF (incidence rate ratio 1.88; 95% CI 1.19-3.05). This differences was statistically significant (P < 0.004) (Table 2).

**Table 2:** Exposure, number of injuries and incidence of injuries on dirt field (DF) and artificial turf field (ATF).

	Exposure, playing hours	Number of injuries	**Injury incidence**, n/1000 playing hours (95% CI)
DF	1897	70	36.9(28.7-46.6) *

ATF	1378	27	19.5(12.9-28.5) *

Total	3275	97	29.6(24.0-36.1)

*Differences P value < 0.004

In terms of severity of injuries in DF cohort most injuries were slight and minimal but in AT cohort the most injuries were mild (Table 3). Incidence of slight and minimal injuries was significantly greater on DF than on ATF (Table 3).

**Table 3:** Incidence of match injuries sustained on dirt field (DF) and artificial turf field (ATF) a function of injury severity

	DF	ATF		
		
Injury severity	Injuries Incidence n/1000 h of exposure (95% CI)	Injuries Incidence n/1000 h of exposure (95% CI)	Rate ratio (95% CI)	P value (Tow sided)
slight (0 days) & Minimal (1-3 days)	13.70(8.95-20.08)	2.90(0.79-7.43)	4.72(1.63-18.61)	0.0009

Mild (4-7 days)	11.07(6.85-16.92)	9.43(5.02-16.13)	1.17(0.56-2.55)	0.66

Moderate (8-28 days)	8.43(4.82-13.69)	5.80(2.50-11.43)	1.45(0.58-3.92)	0.39

Severe (>28 days)	2.10(0.57-5.39)	1.45(0.17-5.24)	1.45(0.38-22.07)	0.35

The most common injured part on ATF was ankle (25.9%) and on DF was knee (24.3%) (Table 4).

**Table 4:** Location of injuries sustained by the subjects who played on dirt field (DF) and artificial turf field (ATF).

		% of total injuries	
		
Injury location		ATF	DF
**Head and Neck**	Head/face/Neck	7.4	5.7

**Upper limbs**	Shoulder/clavicle	3.7	2.9
	
	Upper arm	3.7	0
	
	Elbow, Forearm, Hand	3.7	10.0

**Trunk**	Trunk	3.7	14.3

**Lower limbs**	Hip/groin/thigh	14.8	15.7
	
	Knee	18.5	24.3
	
	Lower leg/Achilles tendon	14.8	10
	
	Ankle	25.9	14.3
	
	Foot/toe	3.7	2.9

**Total**		100	100

The most common injury type in the DF cohort was skin injuries (e.g. abrasion and laceration) and in the AT cohort was sprain and ligament injury followed by haematoma/contusion/bruise.). Laceration and skin lesion was significantly greater on DF than on AT (P < 0.0003) (Table 5). Incidences of other injury types were not significantly greater on DF than on ATF (Table 5). Most injuries sustained by all subjects were acute (ATF89%, DF 91%) and resulted from a player-to-player contact (ATF59.2%, DF 51.4%) (Table 6). Incidence of acute injuries was significantly higher on DF than on ATF(Table 6).

**Table 5:** Characteristics of injuries incurred during matches, Dirt field (DF), Artificial turf field (ATF)

	DF	ATF		
		
Type of injury	Injuries Incidence n/1000 h of exposure (95% CI)	Injuries Incidence n/1000 h of exposure (95% CI)	Rate ratio (95% CI)	P value (Tow sided)
Fractures and bone stress	2.63[0.85-6.15]	1.45[0.17-5.24]	1.81[0.29-19.07]	0.5

Joint (non-bone) and ligament	8.96[5.22-14.34]	6.53[2.98-12.39]	1.37[0.57-3.49]	0.45

Muscle and tendon	2.63[0.85-6.15]	2.90[0.79-7.43]	0.90[0.19-4.57]	0.88

Contusions	5.79[2.89-10.37]	5.07[2.04-10.46]	1.14[0.40-3.47]	0.79

Laceration and skin lesion	16.34[11.10-23.19]	3.62[1.17-8.46]	4.5[1.73-14.83]	0.0003

Other injuries	0.52[0.01-2.93]	0[0-2.67] *	------	

Total	36.9(28.7-46.6)	19.5(12.9-28.5)	1.88 (1.19-3.05)	

(*)one-sided

**Table 6:** The incidence of match injuries with different natures and causes on dirt field (DF) and artificial turf field (ATF).

	DF	ATF		
		
Mechanisms of injuries	Injuries Incidence n/1000 h of match exposure (95% CI)	Injuries Incidence n/1000 h of match exposure (95% CI)	Rate ratio (95% CI)	P value (Tow sided)
**Nature of onset**				

• Acute	32.68[25.05-41.89]	16.69[10.58-25.04]	1.95[1.19-3.31]	0.004

• Gradual	4.21[1.82-8.30]	2.90[0.79-7.43]	1.45[0.38-6.59]	0.56

**Cause of onset**				

• Not any contact	15.8 (10.6-22.5)	7.2 (3.4-13.3)	2.17 1.03-4.99)	0.02

• Contact with another player	18.9 (13.2-26.2)	11.6 (6.6-18.8)	1.63 (0.88-3.15)	0.09

• Contact with the ball	0.5 (0.01-2.9)	0 (0-2.6)*	-----	

• Contact with other objects	1.5 (0.3-4.6)	0.7 (0.01-4)	2.17(0.17-144)	0.55

(*)one-sided

We did not find any significant differences between percentage of injury in different player positions among players who played on DF and those who played on ATF.

In general 64.9% of the injured part was in the right side and 26.8% in the left side (8.2% not applicable). The differences between two groups in this regards was not significant.

About 45.7% of the injuries in DF cohort occurred in the first halftime and 54.3% in the second halftime. On AT 37% of the injuries occurred in the first halftime and 63% in the second halftime. The rate of injury in the second halftime was higher than first halftime in both DF cohort and AT cohort however these differences were not statistically significant.

## Discussion

The major outcome of this study is that the incidence of match injuries of players who play on DF is higher (about two times) than this rate of players who play on ATF. However, this difference was mainly because of acute slight and minimal injuries and laceration and skin lesions.

The overall incidence of match injuries on AT for men in this study was 19.5 that is consistent with values reported by Ekstrand, et al. (2006) [6] for injury rate on AT (19.6). However Ekstrand, et al. [6] only included traumatic match injuries. Match injury incidence rate on AT reported in this study is lower than those reported by Fuller, et al (2007) [4] and Fuller C.(2006) [11]. Fuller, et al (2007) [4] reported a higher rates of football match injuries on AT among American college and university football teams at 25.43. Similarly Fuller C. (2006) reported incidence rate on AT for the FIFA men's U-17 world cup tournaments as 26 [11]. This might be because our subjects were amateur and low level players. The incidence of football injuries reported to be higher in the professional players compare to the low-level players [12,13]. Our reported rate is consistent with those injury rates reported for amateur and low level football players 11.7 to 21.7 [13-17].

We not found equivalent published data to compare results of this study to the incidence of football injury on DFs. The incidence rate of injuries on DF in this study (36.9) was about two-times more than this rate on ATF. This rate (36.9) is higher that reported football match injury rate on gravel at 20 per 1000 hours of game time [2,8]. This rate also is higher than football injury incidence rate on ATF reported by other studies (19.6 - 26) [4,6,11]. Moreover, this rate is higher than football injury incidence rate reported in the literature that is ranged from 11.7 to 35.3 per 1000 hours [9,12,13,18]. This difference was mainly because the rate of slight and minimal injuries was high on DF in the present study. Incidence of slight and minimal injuries was significantly greater on DF (13.70) than on AT (2.90). This rate on DF was higher that rates reported by other studies on AT (4.97) [6] and (8.34) [4].

The stiffness of a surface and the friction between surface and shoe have been reported as important football injury risk factors [2,3], which might explain why the frequency of injury was significantly higher on DF than on ATF in this study.

Uneven surface also might be a possible risk factor for football injuries [19]. However all football fields in this study were not uneven.

Players wear shoes with no spikes at the bottom to play on dirt field but they wear special shoes with spikes to play on artificial turf. More study is needed to evaluate the effects of shoes on injury rates in different fields.

The higher rate of injury and especially slight and minimal injury in this study might be partly because of the employed injury definition. New definition of injury presented by the international consensus statement for epidemiological studies of injuries in football [10] is not exclusively based on time loss or medical attention that might lead to record more of slight and probably minimal injuries. Some abrasion injuries may not lead to time loss and therefore, are not included in some studies. Generation of AT also might affect the incidence of abrasion injuries. Some may discuss that the higher incidence rate of injuries on dirt field was mainly because of laceration and skin lesions and slight and minimal injuries are not as important as more severe injuries. If we do not include these mild injuries the injury rates on dirt field and artificial filed are not significantly different.

In present study the injury incidence on AT was peaked for injuries of mild (4-7 days) severity (9.43) that is in contrast with both Ekstrand et al[6] and Fuller, et al [4] studies. In Ekstrand et al[6] study incidence of injuries was peaked for injuries of moderate (8-28 days) severity (6.35). In Fuller, et al [4] study the incidence of injury decreased with increasing injury severity on both AT and grass. In present study the injury incidence on DF decreased with increasing injury severity.

Joint (non-bone)/ligament injuries to the lower limb was reported as the most common combination of injury type and location sustained on AT in this study. This is similar to results reported in previous studies of football injuries on both grass and AT surfaces [4]. However, in present study on DF laceration and skin lesion was the most common injuries followed by Joint (non-bone)/ligament injuries.

An important cause of football injuries is contact with another player that reported to be varied from 41% to74% [12]. The results of this study are consistent with this data in that most injuries sustained by all subjects were resulted from a player to player contact (AT 59.2%, DF 51.4%).

As a limitation we only recorded match injuries. More study is needed to evaluate the incidence rate and natures of training injuries on DF. As another limitation we did not analysed the content of the DF. Quality of DFs are not the same. DF surface might be variable surface from field to field and climate to climate.

It would be very difficult to find two similar player groups who play on DF and grass because those players who play on DF normally are amateur and in lower age and playing levels in terms of technique and tactic compare to those who play on grass. However as it was mentioned previously we believed the levels of players who played on DF and AT in this study was about the same.

To prevent skin abrasions on dirt fields the skin area that may potentially receive trauma need to be protected. Protective equipments such as sliding pads, long-sleeve shirts, long socks, "biker" shorts might be used to protect the vulnerable exposed areas [20].

Dirt fields should regularly be made re-flat because uneven surface might be a possible risk factor for football injuries on these fields.

## Conclusions

Results of this study suggest that the incidence of match football injuries on DF are higher (about two times) than this rate on AT. This difference was mainly because of acute minimal injuries and skin lesions. Therefore, in regions in which maintaining natural gross is too expensive AT might be an alternative to reduce the rate of football injuries.

## Competing interests

The authors declare that they have no competing interests.

## Authors' contributions

RK and FH contributed to the study concept and, with VZ, the study design. RK, FH and HH were responsible for the acquisition of data. FH and HH contributed to the analysis and interpretation of the data. RK and VZ drafted the manuscript. RK, FH and HH critically revised the manuscript. All of the authors approved the final version of the manuscript submitted for publication.
